# Supplementary material for: Scalable nanoscale positioning of highly coherent color centers in prefabricated diamond nanostructures
Source: Nat Commun. 2025 Nov 6;16:9803. doi: 10.1038/s41467-025-64758-4 (PMC12592383; doi:10.1038/s41467-025-64758-4)
Supplement: Supplementary file 1 — Supplementary Information [file 41467_2025_64758_MOESM1_ESM.pdf]

Supplementary Information for

Scalable nanoscale positioning of highly coherent color centers in prefabricated diamond nanostructures

Sunghoon Kim,[1] Paz London,[1] Daipeng Yang,[1] Lillian B. Hughes,[1] Jeffrey Ahlers,[1] Simon Meynell,[1] William J. Mitchell,[2] Kunal Mukherjee,[3] and Ania C. Bleszynski Jayich[1,*]

[1] *Department of Physics, University of California, Santa Barbara, Santa Barbara, California 93106, USA*

[2] *Nanofabrication Facility, Department of Electrical and Computer Engineering, University of California, Santa Barbara, Santa Barbara, California 93106, USA*

[3] *Department of Materials Science and Engineering, Stanford University, Palo Alto, CA 94305, USA*

Supplementary Section 1. NV NUMBER ESTIMATION VIA MAXIMUM LIKELIHOOD ESTIMATION

To estimate the average number of nitrogen vacancy (NV) centers per pillar, we measure continuous wave-electron spin resonance (CW-ESR) spectra of 121 pillars each for a given pillar size, irradiation dose, and annealing condition. In a given set of 121 pillars, we count the number of NV orientations l for each pillar to measure the probability distribution $P_\lambda^{exp}(l)$, where λ is an average number of NV per pillar to be estimated.

In Supplementary Figure 1, we perform maximum likelihood estimation (MLE) to fit $P_\lambda^{exp}(l)$ to a model distribution $P_\lambda(l)$ and extract λ [1]. We model the probability distribution as

$$P_\lambda(l) = \sum_{n=0}^{\infty} \frac{\lambda^n e^{-\lambda}}{n!} (1/4)^n S(n,l) {}_4P_l, \tag{1}$$

where $S(n,l)$ is the Stirling number of the second kind and ${}_4P_l$ is the number of permutations of l orientations from a total of 4 orientations. This distribution assumes that the number of NVs per pillar follows a Poisson distribution. Also, the units for irradiation dose are in total number of electrons, where $1\,\mathrm{pC} = 2\times10^{18}\,e\,\mathrm{cm}^{-2}$.

Note that the MLE method becomes less precise for very low ($\lambda << 1$) or large ($\lambda >> 16$) average NV number per pillar. We characterize the additional uncertainty due to the systematics of our method by numerically simulating 100 sets of 121 random variables following a Poisson distribution with a given expectation λ_{true}. Then, we perform MLE for each set i to get λ_i, from which we characterize the systematic uncertainty by calculating the standard deviation of the error $\sqrt{\frac{1}{100}\sum_{i=1}^{100}(\lambda_i - \lambda_{true})^2}$. The additional error is added in Figure 2 in the main text.

Supplementary Section 2. SECONDARY ION MASS SPECTROMETRY

SIMS is performed with a CAMECA IMS 7f dynamic instrument using a primary Cs^+ beam energy of 7 kV and current of ∼30 nA at an incident angle of 21.7°. The sample is biased to -3000 V and $^{12}C^{15}N^-$ negative secondary ions are detected using a high mass resolving power, M/ΔM = 6006. Only ions from the central 33 µm are collected from the 100 µm sputtering crater to avoid edge effects. Supplementary Figure 2 shows a SIMS depth profile of the sample studied in this work, highlighting the composition of ^{15}N (collected as a $^{12}C^{15}N^-$ ion, displayed in red) and ^{13}C (black, dashed). The drop in ^{13}C concentration indicates the start of the ^{12}C purified epitaxy, and an unintentional nitrogen peak is seen at this substrate-nitrogen interface. The intentionally δ-doped layer occurs at 53 nm deep and has a thickness of 3.66(2) nm, as determined from the full width at half-maximum (FWHM) of a Gaussian fit to the peak. To calculate the areal density in the 53-nm-deep peak, the peak is integrated over three standard deviations, resulting in $1.736(9)\times10^{12}$ atoms/cm^2 or 98.6(5) ppm·nm. The stated errors represent a 95% confidence interval for a Gaussian peak fit.

To ensure that our results presented in the main text are dominated by the NVs in the 53 nm-deep peak, and not from the interface peak at a depth of 154 nm, we similarly calculate the ^{15}N areal density in the 154-nm-deep peak

* ania@physics.ucsb.edu

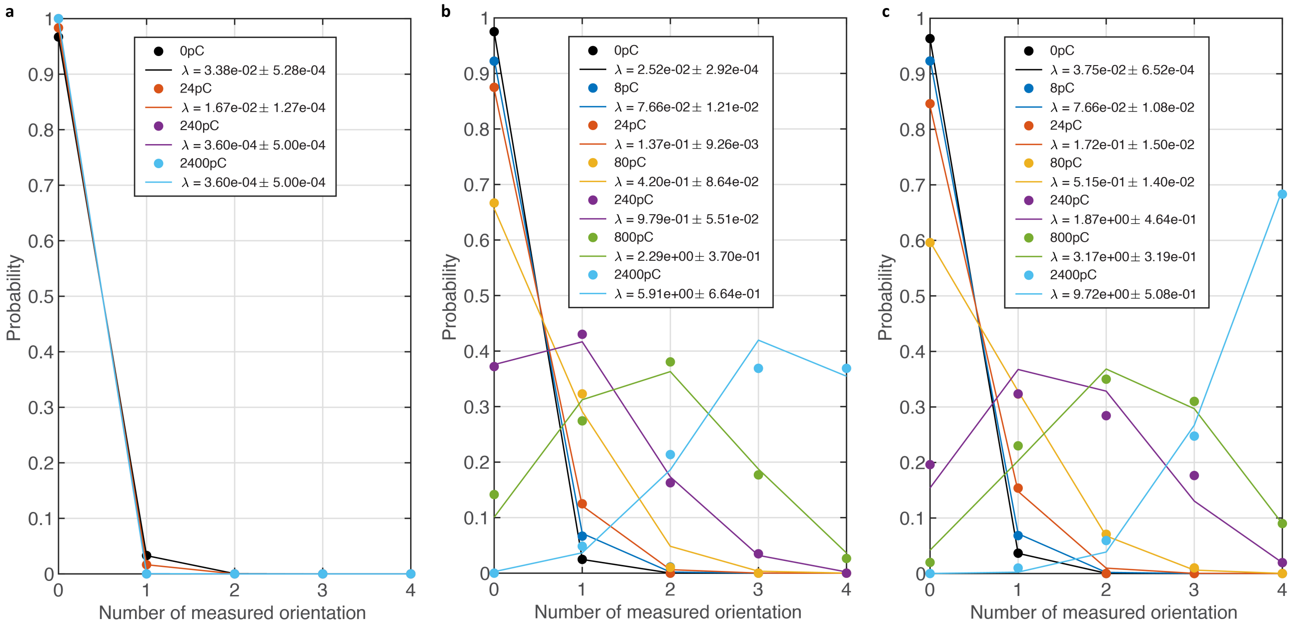

Supplementary Figure 1. Probability distribution of measured NV orientations in a nanopillar. We measure probability distribution (filled circles) of a) non-annealed 480 nm pillars, b) annealed 280 nm pillars, and c) annealed 480 nm pillars for different irradiation doses. Each plot is a result of measuring 121 pillars. We perform MLE to find the best fit to the model probability distribution (solid lines) parameterized by λ, the mean number of NVs per pillar. Indicated errors on λ denote 95 % confidence intervals of the fit.

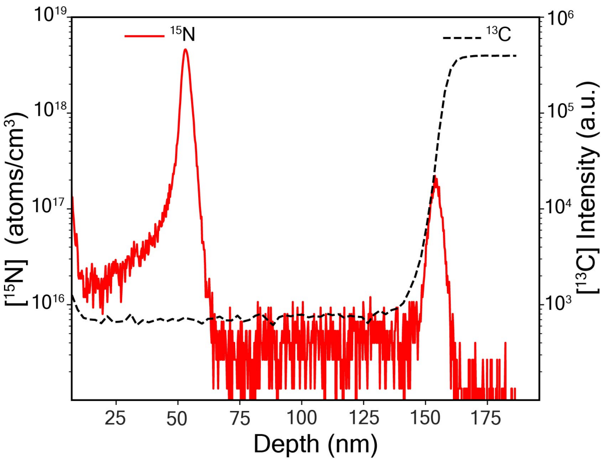

Supplementary Figure 2. SIMS depth profile showing the ^{15}N (collected as a ^{12}C^{15}N^{-} ion) and ^{13}C composition as a function of depth.

and find it to be 5.2 ppm·nm. Hence 94 % of the ^{15}N is in the intentionally-doped 53-nm-deep peak. We also note that when selecting NV centers for measurement, we rule out those which show ^{13}C bath coupling in a Hahn-echo sequence [2].

Supplementary Section 3. VACANCY FORMATION VIA δ-ELECTRON IRRADIATION

We use a 200 keV electron beam with 20 nm diameter spot size (δ-electron irradiation) to create vacancies at the center of the nanopillars. Alignment marks fabricated on the sample allow navigation of the electron beam to the target positions. The alignment accuracy and minimal beam size of the electron irradiation confines the irradiation-induced damaged region of the diamond to the localized position with respect to the prefabricated nanostructure. When its energy exceeds 145 keV, an electron can create a Frenkel defect in the diamond [1]. A Frenkel defect is created by a carbon atom displaced from its original lattice site to create a vacancy site and an interstitial carbon site.

We implement CASINO simulations [3] to characterize vacancy formation via δ-electron irradiation, as shown in Supplementary Figure 3(a). We generate 2 000 000 independent trajectories of electrons with the same energy and spot size as our electron beam in a 2 µm thick diamond. Then, we locate the scattering sites where the energy loss of the electron ΔE due to scattering is greater than the threshold displacement energy $E_d = 35$ eV of diamond [4]; we consider these sites as vacancy sites. In Supplementary Figure 3(b), a total of 340 vacancy sites have been formed, corresponding to 8.5×10^{-5} vacancy/µm electron.

The number of simulated vacancy sites has minimal dependence on depth for the entire range of 2 µm, as shown in Supplementary Figure 3(c) and consistent with ref [5], where vacancy density is depth independent up to a few tens of µm. Another important feature of our simulation is that the vacancy sites remain laterally confined to the spot size throughout the 2 µm depth extent of the simulated diamond, as shown in Supplementary Figure 3(d). This narrow electron trajectory is a result of the vertical momentum of electrons being sufficiently high such that any collisions cannot effectively divert the trajectories of the electrons up to the depth of our simulation.

One can estimate the number and spatial distribution of monovacancies using the simulation with several caveats. First, interstitial carbon atoms form in the same amount as the number of vacancies, where they can recombine during annealing. In another effect, a carbon atom displaced by electron irradiation can create additional vacancies along the trajectory. However, we expect this latter effect to be minimal since the maximum possible energy transfer from a 200 keV electron to a carbon atom is lower than the minimum kinetic energy for a carbon atom to initiate such a cascade effect [6]. Thus, the density of monovacancies is expected to be lower than what we simulate.

In Supplementary Figure 3(e), we also investigate monovacancy creation from different irradiation energies. We analyze 1×10^6 electron trajectories with initial energies 150 keV, 200 keV, and 300 keV, as described above. The distrubutions of the vacancy sites from 150 keV and 300 keV electrons are uniform in z direction, localized within the beam diameter, showing no noticeable difference with 200 keV results. However, the total number of vacancies monotonically increases with energy. Notably, the creation efficiency is nonlinear with energy, with 150 keV electrons showing a five-fold decrease compared to 200 keV. This is due to proximity to the minimum electron energy for monovacancy creation of 145 keV [1] set by a finite E_d.

Supplementary Section 4. MODELING NV FORMATION

The number of created NVs N_{NV} estimated from MC simulations for a given δ-electron irradiation depends on the number of monovacancies N_V, the number of nitrogen N_N within the monovacancy diffusion area, and the probability of capture when a vacancy migrates to a neighboring site of a nitrogen atom p_{cap}. However, while N_N and N_{NV} and can be experimentally measured, N_V and p_{cap} are hard to measure and thus considered free parameters to fit the data. Furthermore, when N_V is sufficiently small, $N_{NV} \propto N_N \cdot p_{cap} \cdot N_V$, which makes it difficult to distinguish their contributions independently. Hence, we define $p_{cap}N_V \equiv \alpha N_V^{max}$, where α is a constant prefactor and N_V^{max} is the maximum number of vacancies calculated from the CASINO simulation.

In Supplementary Figure 4, we characterize the prefactor α from our experimental data. In simulations, we sweep α from 1.2×10^{-2} to 2.4×10^{-1} for different irradiation dose for 280 nm pillars, 480 nm pillars, and mesas and plot the results in open triangles in Supplementary Figure 4 a,c,e. We arrive at a best fit to our data of $\alpha = 0.024(2)$, which we use to plot the simulations in Figure 2 in the main text. This small (<1) value of α can have many origins including nonunity capture probability, Frenkel defect recombination [6, 7] and vacancy cluster formation [8, 9].

Supplementary Section 5. ESTIMATION OF MONOVACANCY DIFFUSION CONSTANT

By comparing the number of NV centers formed in 280 nm diameter pillars N_{NV}^{280} and 480 nm diameter pillars N_{NV}^{480} after $t_{anneal} = 11$ min, one can estimate the monovacancy diffusion constant, as long as the diffusion length is larger than the pillar size, *i.e.* $2\sqrt{2D_V t_{anneal}} \approx \phi/2$, where D_V is monovacancy diffusion constant and ϕ is the pillar

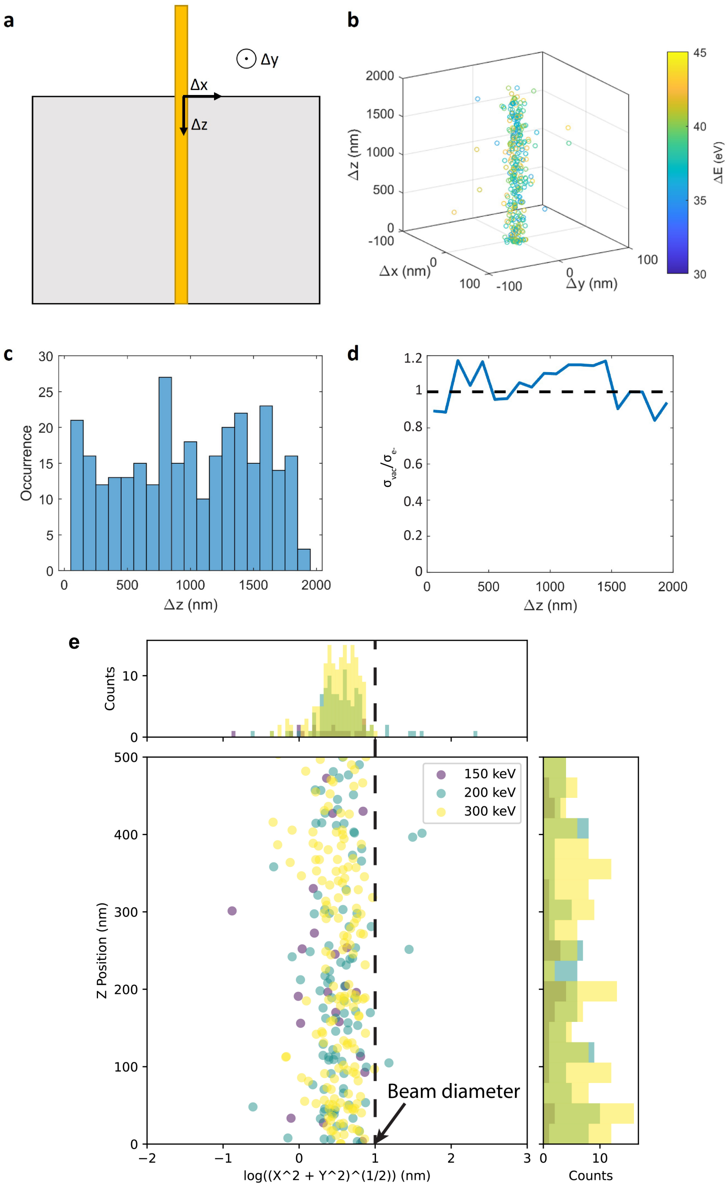

Supplementary Figure 3. CASINO simulation of vacancy creation. (a) Schematic of CASINO simulation. The 200 keV electron beam with a spot size of 20 nm (yellow) is incident on the top surface of a 2 µm thick diamond sample (gray). (b) Vacancy creation sites from 2×10^6 electron trajectories where an electron scatters from a carbon atom and loses kinetic energy of ΔE greater than the displacement energy $E_d = 35\,\mathrm{eV}$. The colors of the markers indicate ΔE. (c) Histogram of the depths of the vacancy sites. (d) The median lateral displacement of the vacancies σ_{vac} as a function of Δz. σ_{vac} is normalized to the size of the electron beam σ_{e^-} (median displacement $\approx 7\,\mathrm{nm}$), where the normalized value of 1 is highlighted with the dotted line. (e) Vacancy creation sites from 1×10^6 electron trajectories for different irradiation energies.

diameter. One must also consider that compared to bulk diamonds, the number of NVs formed inside nanopillars during vacancy diffusion is suppressed due to vacancy absorption at the pillar sidewalls.

In Supplementary Figure 5, we show the relative number of NVs ($\frac{N_{NV}^{480}}{N_{NV}^{280}}$) created for 280 nm and 480 nm diameter pillars (after $t_{anneal} = 11\,\mathrm{min}$). Overall, the larger pillars host a higher number of created NVs, indicating $2\sqrt{2D_V 660\,\mathrm{s}} > 280/2\mathrm{nm}$. Thus, we characterize the lower bound of D_V to be $3.7\,\mathrm{nm^2\,s^{-1}}$.

We also use MC simulations to simulate the relative number of NVs for different D_V, as shown in solid lines in Supplementary Figure 5. We find $13\,\mathrm{nm^2\,s^{-1}} < D_V < 20\,\mathrm{nm^2\,s^{-1}}$ agrees with measured $\frac{N_{NV}^{480}}{N_{NV}^{280}}$ for all irradiation dose.

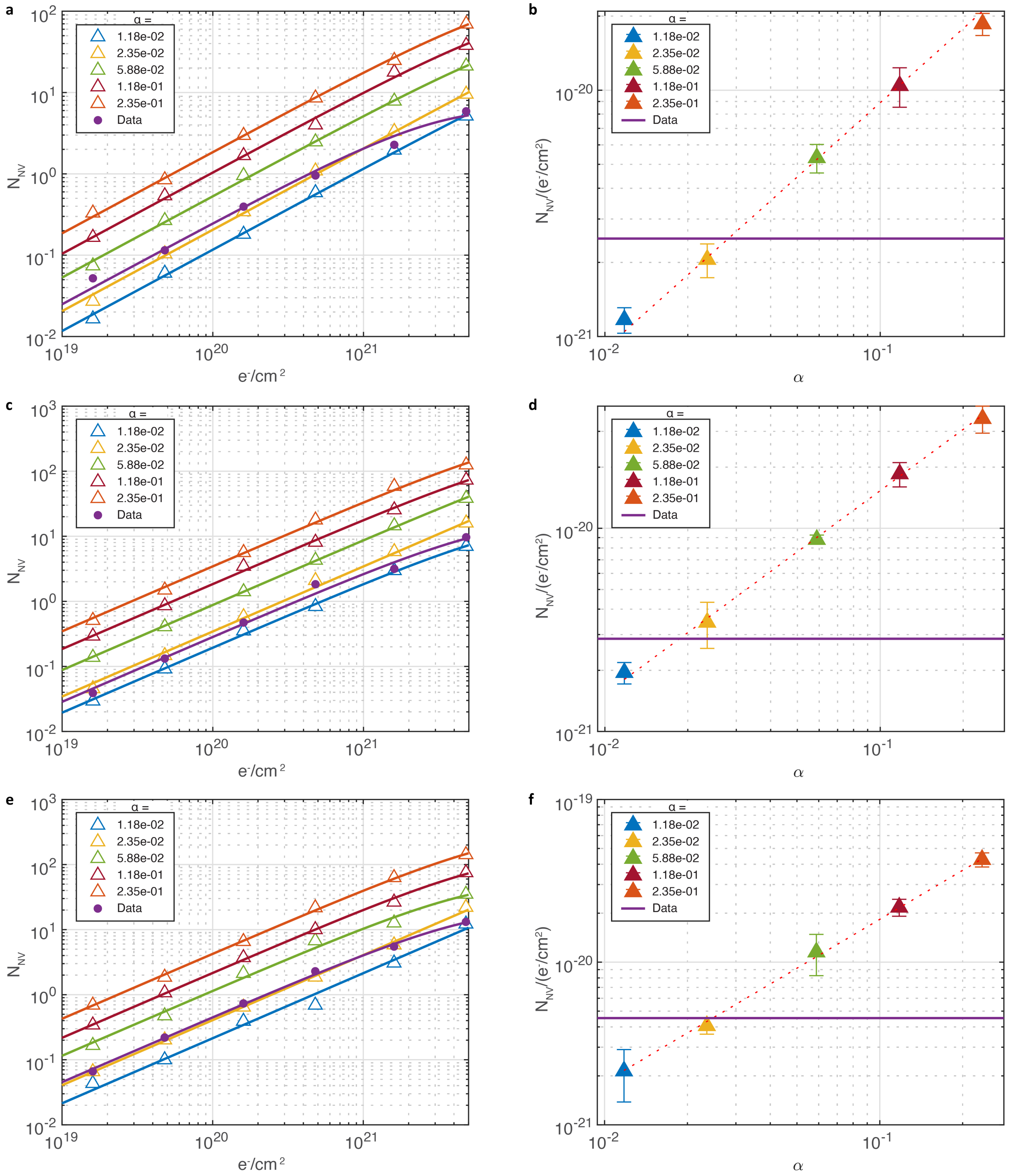

Supplementary Figure 4. Estimating α. Simulated N_{NV} as a function of δ-electron irradiation dose and α for (a) 280 nm pillar, (c) 480 nm pillar and (e) mesa (empty triangles). Our measured N_{NV} for each geometry are shown in purple circles. We fit all data with a function with a linear term and a saturation term [10]. The extracted linear terms for the simulation are shown for (b) 280 nm pillar, (d) 480 nm pillar and (f) mesa (filled triangles, errorbars: 95 % confidence interval). The linear terms are proportional to α, where the mean prefactors for each geometry are plotted in red dotted lines. We use this value to extract α of 0.024(2).

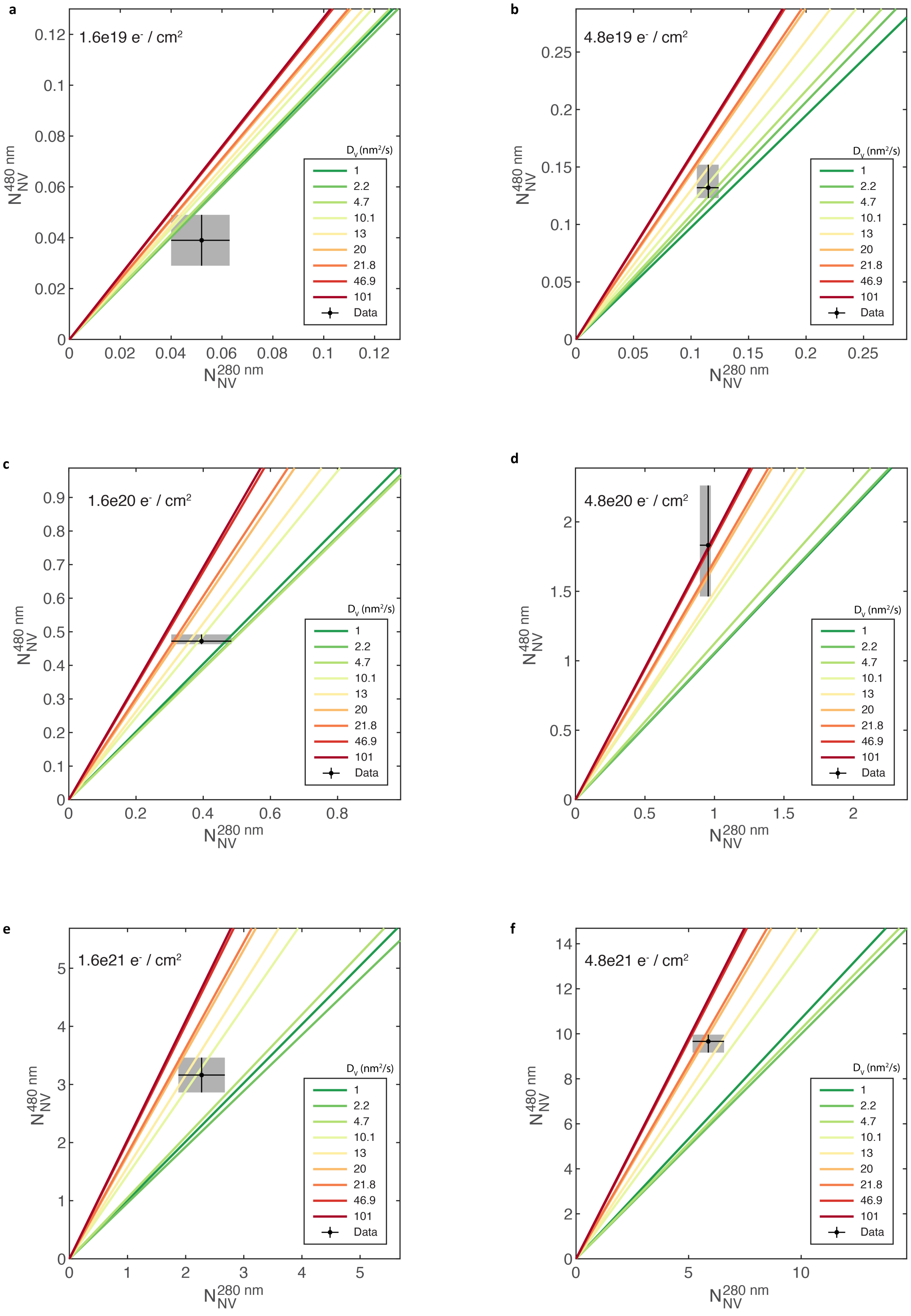

Supplementary Figure 5. Relative number of NVs for two pillar sizes vs. monovacancy diffusion constant. The ratio of the number of NVs for 480 nm pillar to 280 nm pillar $\frac{N_{NV}^{480}}{N_{NV}^{280}}$ is extracted from our MC simulations with varying D_V for (a) 8 pC, (b) 24 pC, (c) 80 pC, (d) 240 pC, (e) 800 pC, and (f) 2400 pC δ-electron irradiation. Solid lines represent $\frac{N_{NV}^{480}}{N_{NV}^{280}}$ for each D_V. Measured values for N_{NV}^{480} and N_{NV}^{280} are plotted with errorbars denoting 95 % confidence interval. The shaded areas show 2-d 90 % confidence intervals for a joint probability distribution of a combination $(N_{NV}^{480}, N_{NV}^{280})$.

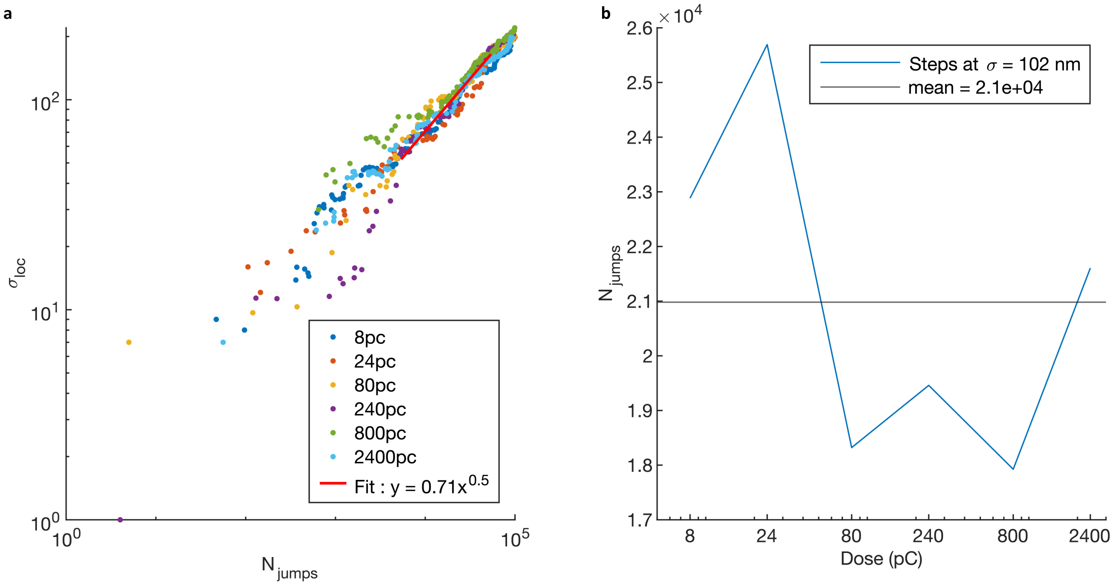

Supplementary Figure 6. Extraction of the total number of monovacancy jumps for a given σ_{loc} (a) Calculated σ_{loc} as a function of the number of jumps. At a large number of jumps, σ_{loc} for all doses shows a power law dependence. (b) The number of jumps at $\sigma_{loc} = 102\,\text{nm}$ is extracted from (a). The distribution of N_{jumps} is characterized by a mean of 2.098×10^4.

Supplementary Section 6. CHARACTERIZATION OF σ_{sys} AND σ_{PSF}

Our method of estimating σ_{loc} requires precise characterization of σ_{PSF} and σ_{sys}. To achieve this, we first perform affine transformations on $40 \times 40\,\mu\text{m}^2$ confocal image to account for aberrations in our imaging system. We use a single image of 2400 pC-irradiated block to optimize the transformations, where the PL maximum positions of the pillars are recorded. We assume the PL maximum positions are set by the spatial profile of the photonic mode of the pillars and the positions of the pillars are the same as the EBL mask design. Then, the lateral displacement of the EBL mask design from the PL maximum positions can be attributed to optical aberrations of the confocal system, where we minimize the root mean square error (RMSE) of the displacements by using affine transformations on the confocal image. Any residual RMSE after optimizing affine transformations is defined as σ_{sys}. We evaluate σ_{sys} by using a different image of 2400 pC-irradiated block, which we find to be 41 nm. After characterizing σ_{sys}, all confocal images taken afterward are transformed using the same transformations.

Then, we characterize σ_{PSF} by measuring the PSF of a single NV in the mesa region. 6 different NVs are identified in the mesa and imaged using our confocal microscopy. The images are then individually fit using a 2D Gaussian function, from which we evaluate $\sigma_{PSF} = 235\,\text{nm}$.

Supplementary Section 7. ESTIMATION OF MONOVACANCY DIFFUSION CONSTANT FROM σ_{loc}

We estimate the monovacancy diffusion constant D_V at 850 °C by directly comparing σ_{loc} with MC simulation results. In three-dimensional diffusion, the diffusing object can be modeled as a random walker that jumps along the edges of a cubic lattice with a lattice constant a and a total number of jumps N_{jumps}^{tot} for a total time t_{anneal}. Then, the diffusion constant is given as $D = \frac{a^2 N_{jumps}^{tot}}{6 t_{anneal}}$.

In Supplementary Figure 6, we use MC simulations with a simplified model for monovacancy diffusion, where monovacancies perform random walks in a cubic lattice with a lattice constant of 2 nm. We collect NV formation events to extract σ_{loc} for a given number of jumps. Then, we use σ_{loc} characterized from our annealed mesas to estimate N_{jumps}^{tot} for $t_{anneal} = 11\,\text{min}$. Lastly, we use the above equation to arrive at $D = 21\,\text{nm}^2\,\text{s}^{-1}$.

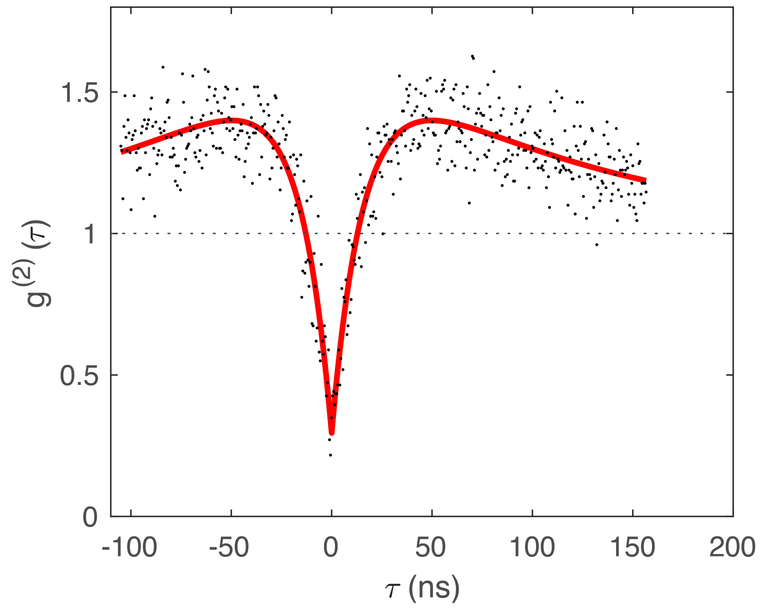

Supplementary Figure 7. A representative second-order autocorrelation function $g^{(2)}(\tau)$ of a single-NV containing pillar.

Supplementary Section 8. SECOND-ORDER AUTOCORRELATION FUNCTION MEASUREMENTS

We measure the second-order autocorrelation function $g^{(2)}(\tau)$ of the nanopillars and identify single-NV pillars as those for which $g^{(2)}(0) < 0.5$, where each $g^{(2)}(\tau)$ is individually fit with antibunching and bunching terms [11]. No background subtraction was performed on the data. Supplementary Figure 7 shows a representative $g^{(2)}(\tau)$ data for one of the single-NV containing pillars presented in Figure 4 in the main text.

Supplementary Section 9. DIPOLE INTERACTION LIMIT OF T_2^{Hahn}

To understand the source of decoherence in our δ-doped samples, we first consider the contribution of substitutional nitrogen (P1) spins on Hahn echo coherence time T_2^{Hahn}. We consider Ising interactions between P1 spins and the NV center and perform averaging over the ensemble of spin state trajectories and positional randomness [12]. To ensure configurational averaging over many NV center P1 environments (our T2 measurements are all on single NVs), we average multiple single NV Hahn-echo decays together. Given the estimated P1 density of 17 ppm $\cdot$ nm from SIMS (the P1 density is typically ~ 6 times less than the nitrogen content determined by SIMS [13]), we calculate the P1-limited T_2^{Hahn} for an ensemble of NVs to be $70 \sim 90\,\mu$s, for a typical correlation times of the P1 spins of $1 \sim 2$ ms [13].

Supplementary Section 10. SATURATION PHOTON COUNT MEASUREMENT

We measure PL_{sat} of nanopillars with single NVs by recording the photon counts PL as a function of the excitation power P_{exc}. Then, we fit the data with the following:

$$PL(P_{exc}) = \frac{PL_{sat}}{1 + PL_{sat}/\alpha_{NV} P_{exc}} + \alpha_{bg} P_{exc}, \tag{2}$$

where α_{NV} and α_{bg} are linear coefficients. The first term concerns PL from the NV and the second term concerns PL from the background. In Supplementary Figure 8, we show a typical PL_{sat} measurement data of a single NV pillar (black circles), from which we extract PL_{sat} of 969.3 kcps. Note that we extract background counts from the fit, which assumes that the power needed to saturate the background is high compared to the NV. However, we do not see additional non-linear behavior besides the NV term up to the highest P_{exc} that we use, implying that the background is effectively linear.

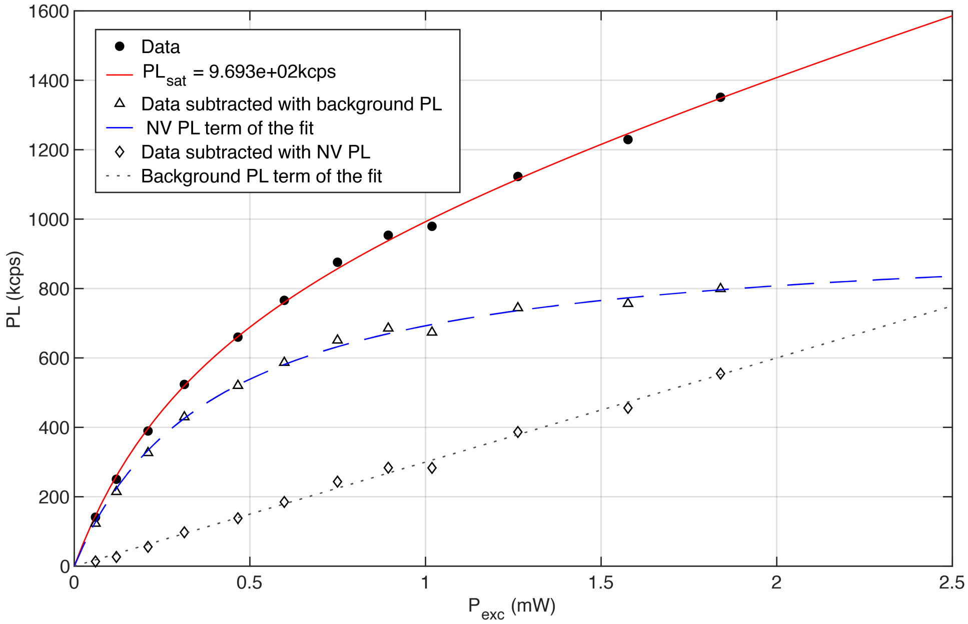

Supplementary Figure 8. Photon count rate of a typical single NV pillar as a function of excitation power. The data (black circles) are plotted with the fit curve (red solid line), which is a sum of an NV PL term (blue dashed line) and a background PL term (gray dotted line). We also show the data subtracted with each of the two terms with open triangles and open diamonds.

Supplementary Section 11. FINITE-DOMAIN TIME-DEPENDENT SIMULATION RESULTS

We perform FDTD simulations to calculate the photon collection efficiency for a given NV lateral displacement. First, we characterize the geometries of the pillars with top diameter ϕ_{top} of 480 nm and 280 nm from scanning electron micrographs. The nanopillars do not have perfectly vertical sidewalls from the dry etch. We measure height h of 1414 µm with bottom diameter ϕ_{bottm} of 850 nm and 610 nm for 480 nm and 280 nm pillars, respectively. We use these values in the FDTD simulations described in the main text.

Supplementary Figure 9 shows the calculated collection efficiency of the nanopillars as a function of the NV lateral displacement from the pillar axis. The depth of the dipole is fixed at 53 nm and the NV orientation is set to $[0, \sqrt{2}, 1]$ with respect to the FDTD coordinate system. In Supplementary Figure 9 (a-d), we show the collection efficiency of 280 nm pillar as a function of lateral displacement dx and dy for a given emission wavelength and dipole orientation. We then average over the wavelength and dipole orientation to calculate collection efficiency for a given NV lateral displacement, as shown in Supplementary Figure 9 (e). Supplementary Figure 9 (f) shows the full 2D map of the collection efficiency for an arbitrary lateral displacement inside the 280 nm pillar extrapolated from Supplementary Figure 9 (e), as described in the main text. Likewise, we perform the same simulations for 480 nm pillars, as shown in Supplementary Figure 9 (g-j), to create the same 2D map in Supplementary Figure 9 (l).

We then use the truncated Gaussian function with a spread of σ_0 to calculate the mean collection efficiency as a function of σ_{loc}^{pillar} as described in the main text.

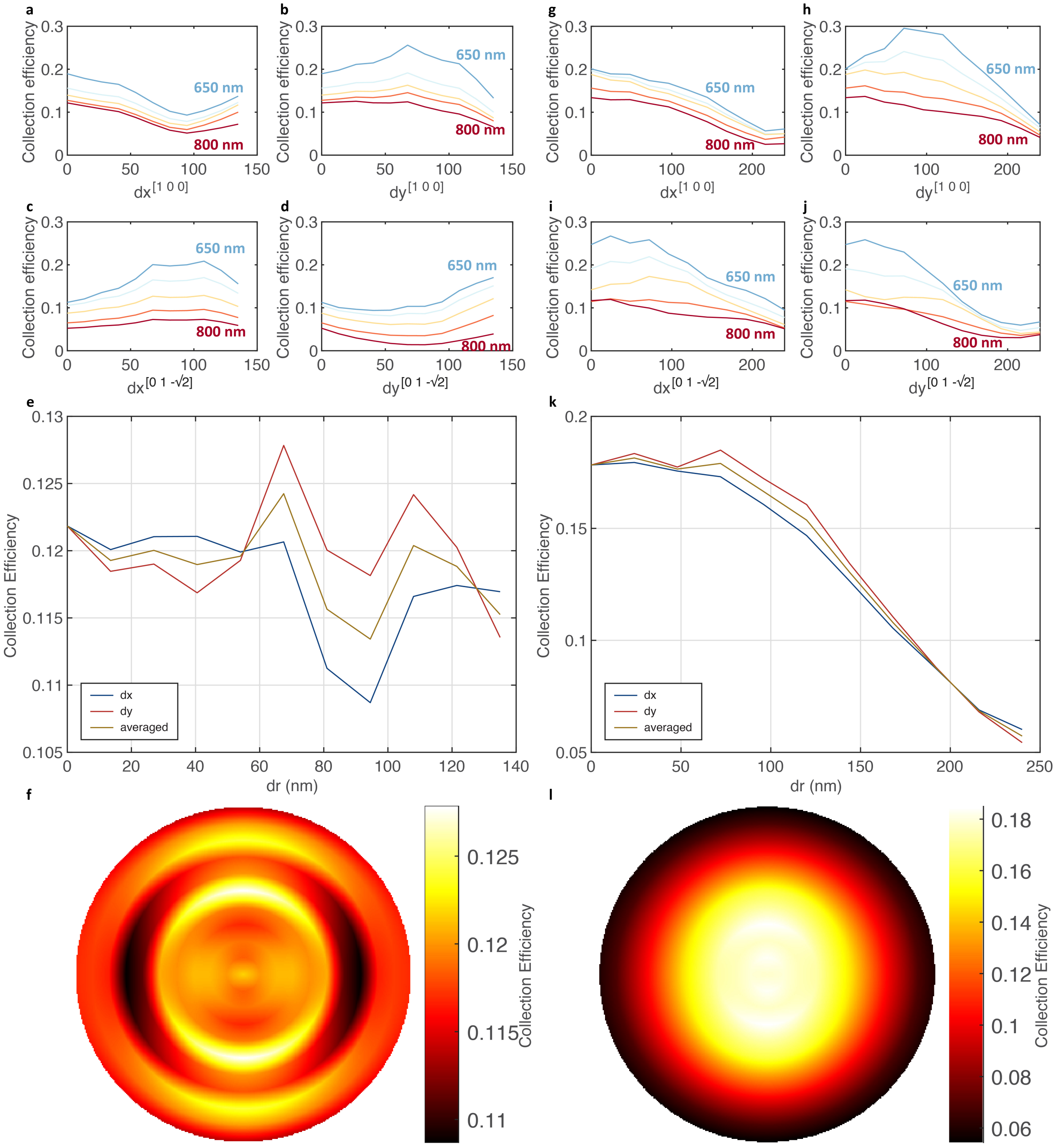

Supplementary Figure 9. Collection efficiency of nanopillars as a function of NV lateral displacement. (a-d) The collection efficiency of 280 nm pillar for a given dipole orientation orthogonal to the NV axis is calculated for each emission wavelength for a given orthogonal displacements dx and dy. (e) For each displacement in the 280 nm pillar, we calculate the collection efficiency of an NV by performing a weighted average over the wavelengths and then averaging the two dipole orientations. (f) The 2D map of a collection efficiency inside 280 nm pillar is constructed from (e) as described in the main text. (g-j) Same as (a-d) but for 480 nm pillar. (k) Same as (e) but for 480 nm pillar. (l) Same as (f) but for 480 nm pillar.

[1] C. A. McLellan, B. A. Myers, S. Kraemer, K. Ohno, D. D. Awschalom, and A. C. Bleszynski Jayich, Patterned Formation of Highly Coherent Nitrogen-Vacancy Centers Using a Focused Electron Irradiation Technique, Nano Letters **16**, 2450 (2016), publisher: American Chemical Society.

[2] L. Childress, M. V. Gurudev Dutt, J. M. Taylor, A. S. Zibrov, F. Jelezko, J. Wrachtrup, P. R. Hemmer, and M. D. Lukin, Coherent Dynamics of Coupled Electron and Nuclear Spin Qubits in Diamond, Science **314**, 281 (2006), publisher: American Association for the Advancement of Science.

[3] D. Drouin, A. R. Couture, D. Joly, X. Tastet, V. Aimez, and R. Gauvin, enCASINO V2.42—A Fast and Easy-to-use Modeling Tool for Scanning Electron Microscopy and Microanalysis Users, Scanning **29**, 92 (2007), _eprint: https://onlinelibrary.wiley.com/doi/pdf/10.1002/sca.20000.

[4] J. C. Bourgoin and B. Massarani, Threshold energy for atomic displacement in diamond, Physical Review B **14**, 3690 (1976), publisher: American Physical Society.

[5] B. Campbell and A. Mainwood, enRadiation Damage of Diamond by Electron and Gamma Irradiation, physica status solidi (a) **181**, 99 (2000).

[6] E. Losero, V. Goblot, Y. Zhu, H. Babashah, V. Boureau, F. Burkart, and C. Galland, Creation of NV centers in diamond under 155 MeV electron irradiation (2023), arXiv:2305.15009 [physics, physics:quant-ph].

[7] D. Antonov, T. Häußermann, A. Aird, J. Roth, H.-R. Trebin, C. Müller, L. McGuinness, F. Jelezko, T. Yamamoto, J. Isoya, S. Pezzagna, J. Meijer, and J. Wrachtrup, Statistical investigations on nitrogen-vacancy center creation, Applied Physics Letters **104**, 012105 (2014).

[8] M. Kasperczyk, J. A. Zuber, A. Barfuss, J. Kölbl, V. Yurgens, S. Flågan, T. Jakubczyk, B. Shields, R. J. Warburton, and P. Maletinsky, enStatistically modeling optical linewidths of nitrogen vacancy centers in microstructures, Physical Review B **102**, 075312 (2020).

[9] S. Santonocito, A. Denisenko, R. Stöhr, W. Knolle, M. Schreck, M. Markham, J. Isoya, and J. Wrachtrup, enNV centres by vacancies trapping in irradiated diamond: experiments and modelling, New Journal of Physics **26**, 013054 (2024), publisher: IOP Publishing.

[10] S. T. Alsid, J. F. Barry, L. M. Pham, J. M. Schloss, M. F. O'Keeffe, P. Cappellaro, and D. A. Braje, enPhotoluminescence Decomposition Analysis: A Technique to Characterize N - V Creation in Diamond, Physical Review Applied **12**, 044003 (2019).

[11] M. Berthel, O. Mollet, G. Dantelle, T. Gacoin, S. Huant, and A. Drezet, Photophysics of single nitrogen-vacancy centers in diamond nanocrystals, Physical Review B **91**, 035308 (2015), publisher: American Physical Society.

[12] E. J. Davis, B. Ye, F. Machado, S. A. Meynell, W. Wu, T. Mittiga, W. Schenken, M. Joos, B. Kobrin, Y. Lyu, Z. Wang, D. Bluvstein, S. Choi, C. Zu, A. C. B. Jayich, and N. Y. Yao, enProbing many-body dynamics in a two-dimensional dipolar spin ensemble, Nature Physics **19**, 836 (2023), publisher: Nature Publishing Group.

[13] L. B. Hughes, Z. Zhang, C. Jin, S. A. Meynell, B. Ye, W. Wu, Z. Wang, E. J. Davis, T. E. Mates, N. Y. Yao, K. Mukherjee, and A. C. Bleszynski Jayich, Two-dimensional spin systems in PECVD-grown diamond with tunable density and long coherence for enhanced quantum sensing and simulation, APL Materials **11**, 021101 (2023).
